# Isomerization of spiropyran photoswitches in microphase-separated block copolymers

**DOI:** 10.1080/14686996.2025.2590800

**Published:** 2025-11-20

**Authors:** Keiichi Imato, Koki Momota, Ichiro Imae, Tomoyasu Hirai, Yousuke Ooyama

**Affiliations:** a Applied Chemistry Program, Graduate School of Advanced Science and Engineering, Hiroshima University, Higashihiroshima, Japan; b Department of Applied Chemistry, Faculty of Engineering and Graduate School of Engineering, Osaka Institute of Technology, Osaka, Japan

**Keywords:** Photoswitch, spiropyran, isomerization, block copolymer, microphase separation

## Abstract

Molecular photoswitches are incorporated into materials to impart photoresponsiveness, enabling a wide range of fascinating applications. Although their surrounding environments within materials strongly influence the photoresponsive and thermally reversible behaviors through physical and chemical interactions, these effects remain poorly understood. In this study, we investigate the two-way photoisomerization and thermal back-isomerization of spiropyran (SP)—a representative photoswitch that undergoes large polarity changes upon the reversible isomerization to merocyanine (MC)—chemically integrated into diblock copolymers (dBCPs) exhibiting either ordered or disordered microphase separation. We synthesize a series of dBCPs consisting of a high-glass-transition-temperature (*T*_g_) poly(methyl methacrylate) block and a low-*T*_g_ statistical copolymer block of SP acrylate and *n*-butyl acrylate, with varying compositions, molecular weights, and microphase-separated structures. The results reveal that the rates of both the SP-to-MC and MC-to-SP photoisomerization processes differ substantially between ordered and disordered microphase-separated structures but are comparable among the different ordered morphologies. In contrast, the photoisomerization yields are scarcely affected by the microphase separation. These findings provide valuable insights into the molecular and polymer design of photoresponsive smart materials based on photoswitches and will contribute to their further development and applications.

## IMPACT STATEMENT

Isomerization of spiropyran photoswitches chemically incorporated into diblock copolymers exhibiting either ordered or disordered microphase separation is investigated, demonstrating that the microphase separation can control the photoisomerization rates.

## GRAPHICAL ABSTRACT

**Figure:**
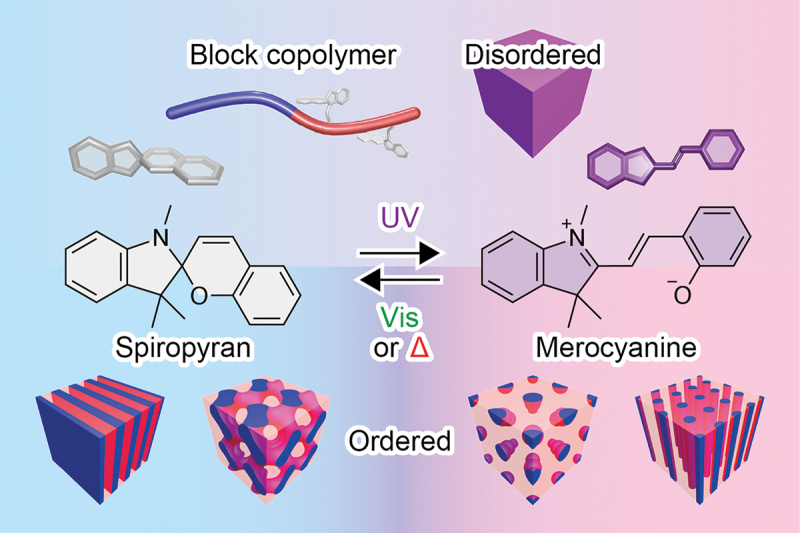

## Introduction

Molecular photoswitches (thermo)reversibly isomerize between at least two thermodynamically (meta)stable states under photoirradiation in a wavelength-selective manner and have been attracted widespread attention owing to their unique advantages including photostimulation, such as the reversibility, production of no chemical waste, high spatiotemporal resolution, rapid and non-contact way, and easy and precise control of wavelength and intensity [1–14]. A variety of photoswitches are still being developed and have been incorporated into materials to impart photoresponsiveness, enabling fascinating applications, e.g. information encryption, light-driven actuators and healing, photolithography, solar energy storage, and photoswitchable adhesives, catalysts, cell culture scaffolds, and vehicles [1–14]. However, the surrounding environments of photoswitches within materials strongly affect the photoresponsive and thermally reversible behaviors through physical and chemical interactions and often degrade the photoresponsive properties, i.e. photoisomerization rate and (quantum) yield, but improve the thermally reversible properties, i.e. thermal stability (half-life). Therefore, the photoisomerization and thermal back-isomerization of photoswitches chemically or physically integrated into bulk materials and thin films and on surfaces have been studied intensively in recent years but remain poorly understood [15–17].

**Figure 1.**
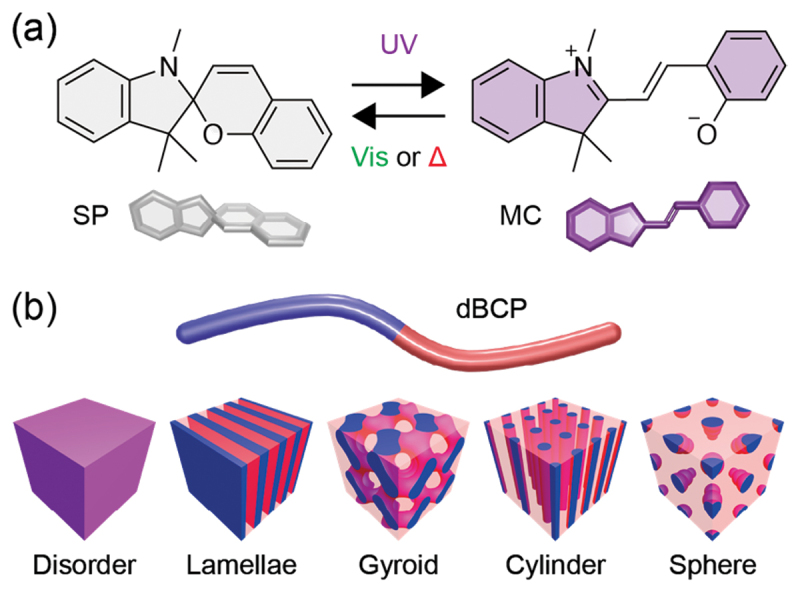
(a) Photoisomerization and thermal isomerization between SP and MC. (b) Microphase-separated structures of dBCPs.

In this study, we focused on spiropyran (SP) and microphase separation of block copolymers (BCPs) (Figure 1). SP is a representative photoswitch following azobenzene [18–35] and characterized by the large polarity changes (Figure 1(a)) [36,37]. Under UV light, weakly polar (dipole moment (*μ*) = 4–6 D), bulky, noncharged SP isomerizes to highly polar (*μ* = 14–18 D), planar, zwitterionic merocyanine (MC), which reverts back to SP spontaneously or under visible light. BCPs are composed of chemically distinct, immiscible polymer segments, phase-separate, and self-assemble into various periodic nanostructures, which have been widely used in diverse materials and applications, such as thermoplastic elastomers, electronics, photonics, nanotemplates, and nanomaterial ordering (Figure 1(b)) [38–43]. The morphology, domain size, orientation, and structural integrity are determined by many factors, including the molecular weight and architecture, block volume fraction, interactions between the blocks, and environmental and annealing conditions. Not only SP but also other photoswitches have been incorporated into BCPs, but the photoresponsive and thermally reversible behaviors have not been studied in detail. Here we investigate the photoisomerization and thermal back-isomerization of SP chemically incorporated into diblock copolymers (dBCP) exhibiting either ordered or disordered microphase separation.

## Synthesis and characterization of dBCPs

**Figure 2.**
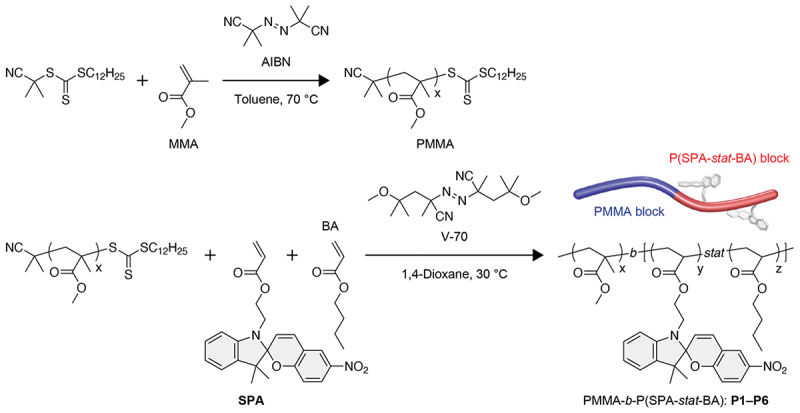
Synthetic route for PMMA-*b*-P(SPA-*stat*-BA).

An SP monomer with a polymerizable acrylate group, **SPA**, was prepared according to our previously reported method [44]. dBCPs bearing SP molecules in the side chains of one block (PMAA-*b*-P(SPA-*stat*-BA)) were synthesized by reversible addition–fragmentation chain-transfer (RAFT) polymerization of methyl methacrylate and subsequent copolymerization of **SPA** and *n*-butyl acrylate (BA) (Figure 2). In other words, the dBCPs were composed of a poly(methyl methacrylate) (PMMA) block and a statistical copolymer (P(SPA-*stat*-BA)) block. We obtained six dBCPs, **P1–P6**, with different compositions, number average molecular weights (*M*_n_s), and degrees of polymerization (DPs) (Table 1), which were calculated based on the ^1^H NMR spectra (Figure S3). The unimodal curves of the PMMA blocks and dBCPs with relatively narrow dispersities (*M*_w_/*M*_n_s) in size exclusion chromatography (SEC) indicate that the RAFT polymerizations were controlled successfully (Figure S2 and S4).

**Table 1.** Compositions, *M*_n_s, DPs, *M*_w_/*M*_n_s, *T*_g_s, and morphologies of **P1**–**P6**.

	*M*_n_/g mol^−1^ (Ratio)			DP (Ratio)			*M*_w_/*M*_n_^*b*^	*T*_g_/°C^*c*^		Morphology^*d*^
	PMMA^*b*^	P(SPA-BA)^*a*^	Total	MMA^*b*^	SPA^*a*^	BA^*a*^		PMMA	P(SPA-BA)	
**P1**	22,000 (1)	51,600 (2.35)	73,900	220 (4.8)	46 (1)	257 (5.6)	1.36	112	9	Cylindrical
**P2**	25,300 (1)	39,600 (1.57)	65,200	253 (7)	46 (1)	257 (5.4)	1.33	109	11	Cylindrical
**P3**	25,300 (1)	43,600 (1.72)	69,200	253 (6.4)	40 (1)	213 (5.4)	1.42	116	5	Cylindrical
**P4**	26,200 (1)	29,500 (1.13)	56,000	262 (9.5)	28 (1)	141 (5.1)	1.33	120	5	Lamellar
**P5**	26,200 (1)	41,300 (1.58)	67,800	262 (7.3)	36 (1)	208 (5.8)	1.35	120	4	Cylindrical
**P6**	36,100 (1)	51,600 (1.43)	88,000	361 (6.7)	54 (1)	232 (4.3)	1.38	121	13	Lamellar

^*a*^Calculated based on ^1^H NMR. ^*b*^Obtained using SEC. ^*c*^Measured by DSC. ^*d*^Determined from SAXS.

## Characterization of microphase-separated structures

**Figure 3.**
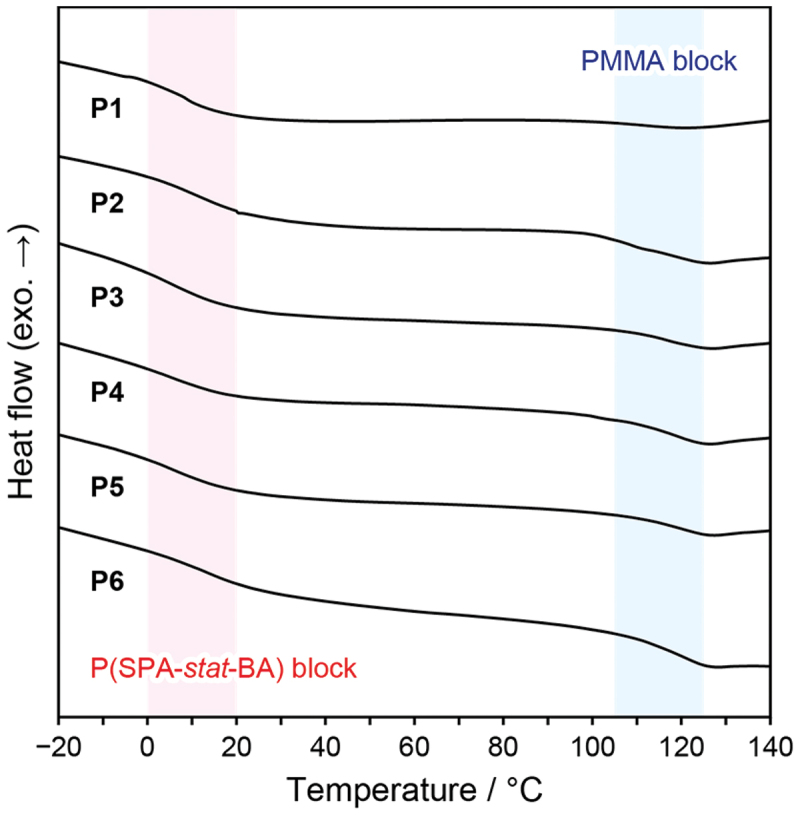
DSC curves of **P1**–**P6**.

All dBCPs clearly showed two distinct glass transition temperatures (*T*_g_s) in the differential scanning calorimetry (DSC) curves, indicating microphase separation of the two blocks (Figure 3). Melting points (*T*_m_s) were not observed in the dBCPs. The *T*_g_s above 100°C and those at approximately 10°C originated from the PMMA blocks and the P(SPA-*stat*-BA) blocks, respectively (Table 1). The *T*_g_s of the corresponding homopolymers for the P(SPA-*stat*-BA) blocks, PSPA and PBA, were reported to be 132°C and below −40°C, respectively [44,45]. Therefore, the major component of PBA significantly deceased the *T*_g_s of the blocks to below room temperature.

**Figure 4.**
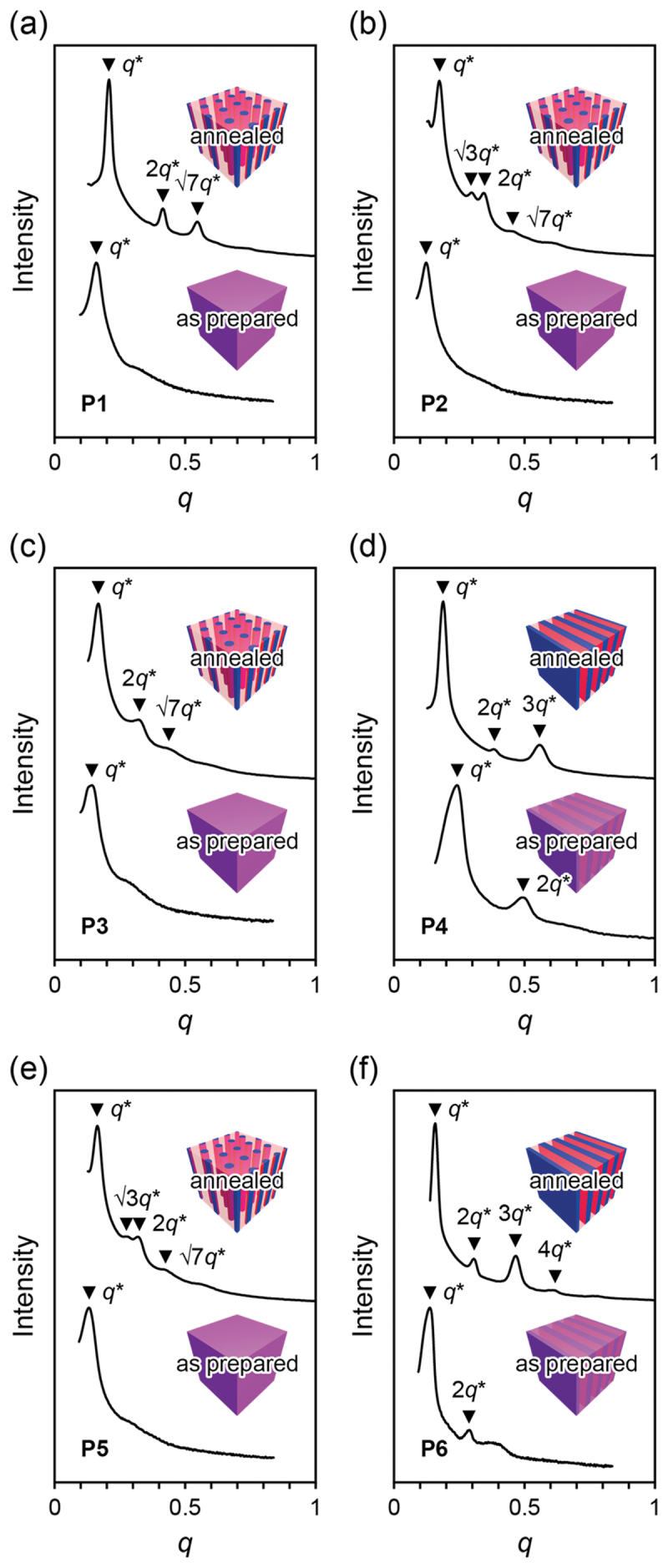
SAXS patterns of as-prepared and annealed (a) **P1**, (b) **P2**, (c) **P3**, (d) **P4**, (e) **P5**, and (f) **P6**.

Microphase-separated structures of the bulk dBCPs were investigated by small angle X-ray scattering (SAXS). We obtained the SAXS patterns of the six dBCPs before and after annealing at 180°C for 16 h under vacuum (Figure 4), where *q* is the scattering vector (*q* = (4π/*λ*)sin*θ*, where 2*θ* is the Bragg angle) and q* is the position of the first-order diffraction peak. As-prepared **P1**–**P3** and **P5** without annealing showed only the first-order diffraction peak, indicating phase-separated but disordered phases. **P4** and **P6** without annealing exhibited broad and diffuse higher-order diffraction peaks. The peak positions suggest lamellar morphologies, which is the most readily formed in the microphase-separated structures [46]. On the other hand, well-defined higher-order diffraction peaks were observed in all dBCPs after annealing. The peak positions indicate that **P1**–**P3** and **P5** showed hexagonally packed cylindrical morphologies (1, √3, 2, √7, 3, √12, √13, …) while **P4** and **P6** exhibited lamellar morphologies (1, 2, 3, 4, …).

## Photoisomerization

### SP to MC under UV light

**Figure 5.**
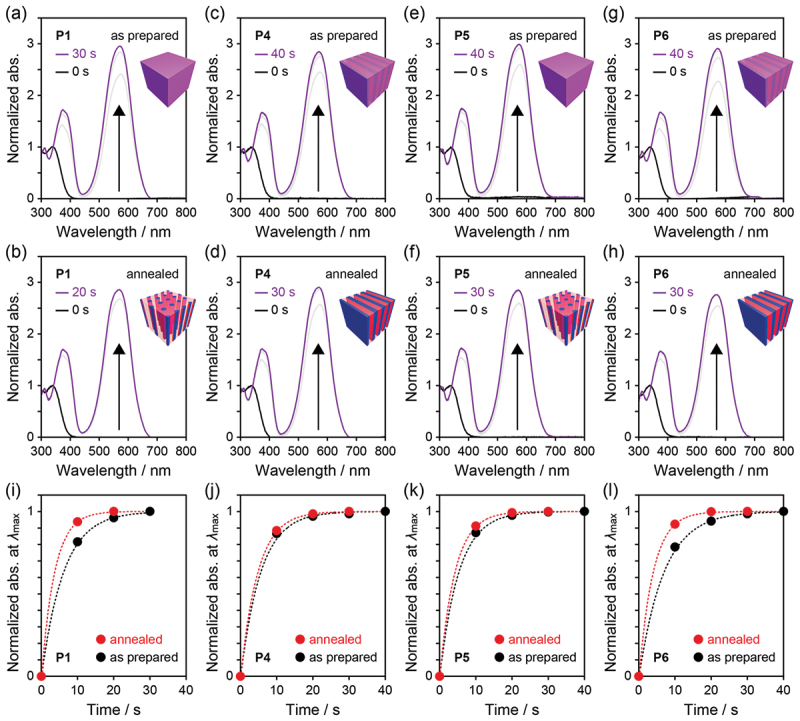
UV–vis absorption spectra of as-prepared and annealed thin films of (a, b) **P1**, (c, d) **P4**, (e, f) **P5**, and (g, h) **P6** under 365 nm irradiation. The spectra are normalized at *λ*_max_ around 340 nm before irradiation. (i–l) Time dependence of normalized absorbance at *λ*_max_ above 450 nm of the thin films under 365 nm irradiation.

**Figure 6.**
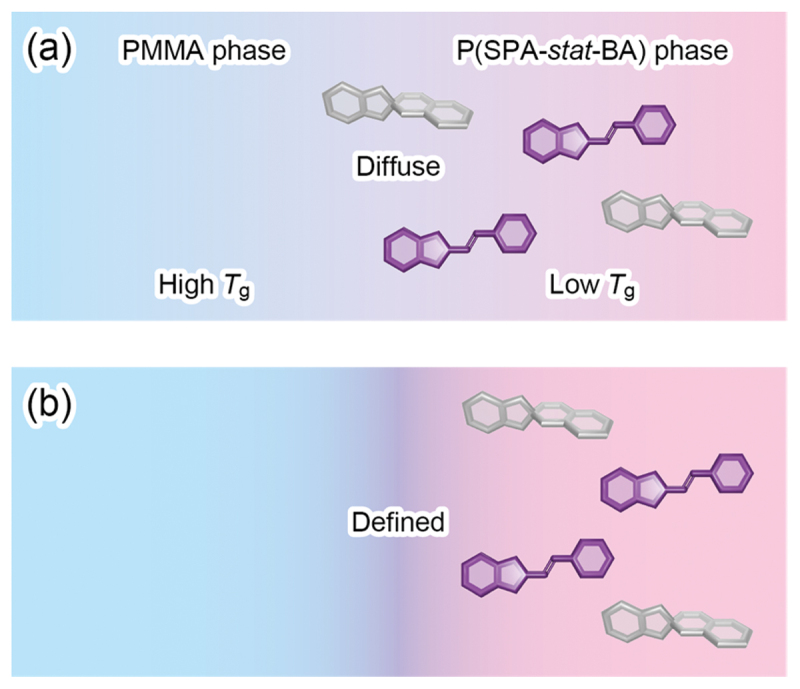
Schematic illustration of surrounding matrix around SP and MC molecules in (a) diffuse (as-prepared) and (b) well-defined (annealed) microphase-separated structures.

The effect of microphase-separated structures on photoisomerization of the incorporated SP molecules was examined in four dBCPs, **P1** and **P4**–**P6**, by UV–vis absorption spectroscopy on the thin films before and after annealing at 180°C for 16 h under vacuum. The thin films with approximately 100 nm thickness were prepared by spin coating of the dBCPs on quartz glass substrates whose surfaces were modified with a hydrophobic monolayer using 1,1,1,3,3,3-hexamethyldisilazane (HMDS). First, we irradiated the thin films with 365 nm UV light. In Figure 5(a–h), the UV–vis absorption spectra were normalized at the maximum absorption wavelength (*λ*_max_) of SP around 340 nm before irradiation. In all four dBCPs, an absorption band peaked at approximately 580 nm appeared and increased with irradiation time, indicating isomerization to MC [36,37,44,47,48]. Although the photoisomerization ratio (maximum absorbance at ca. 580 nm) was independent of the microphase-separated structures, the rate was affected by whether the structures were ordered or disordered. The absorbance at approximately 580 nm was normalized by its maximum value and plotted as a function of irradiation time, yielding kinetic curves (Figure 5(i–l)). These curves were well described by first-order kinetics (*A*(*t*) = 1−exp(−*kt*), where *A*, *k*, and *t* donate the absorbance at ca. 580 nm, the rate constant, and the irradiation time, respectively) [49–51]. The obtained *k* values are summarized in Table 2. The SP-to-MC photoisomerization proceeded more rapidly in the ordered (annealed) films than in the disordered (as-prepared) films. This is probably because the well-defined microphase separation enhanced the molecular mobility of the low-*T*_g_ P(SPA-*stat*-BA) block through distinct segregation from the high-*T*_g_ PMMA block. More specifically, the local free volume surrounding the incorporated SP molecules was larger in the well-defined microphase-separated structures than in the diffuse ones (Figure 6). On the other hand, the morphology type (cylindrical or lamellar) hardly affected either the photoisomerization ratio or the rate. Moreover, no clear correlations were found between the *k*s and the compositions or *M*_n_s of the dBCPs.

**Table 2.** Rate constants (*k*s) of as-prepared and annealed thin films of **P1**, **P4**, **P5**, and **P6** for SP-to-MC photoisomerization under 365 nm irradiation, MC-to-SP photoisomerization under 525 nm irradiation, and MC-to-SP thermal isomerization at room temperature in the dark.

	*k* under 365 nm (SP → MC)/s^−1^		*k* under 525 nm (MC → SP)/10^−3^ s^−1^		*k* at room temperature (MC → SP)/10^−5^ s^−1^
	as prepared	annealed	as prepared	annealed	annealed
**P1**	0.164	0.277	5.08	9.34	9.49
**P4**	0.180	0.210	4.15	6.49	7.59
**P5**	0.189	0.239	3.11	4.09	7.96
**P6**	0.144	0.254	4.03	5.67	7.36

Prolonged UV light irradiation caused a blue shift of the *λ*_max_ and a decline of the MC band (Figure S6), which are attributed to the formation of H-aggregates of MC and degradation of both SP and MC [36,52]. Therefore, the 365 nm irradiation time was fixed at 20–40 s in the subsequent experiments. Additionally, the SP-to-MC isomerization did not accompany any changes in the microphase-separated structures despite the significant increase in the polarity, as confirmed by digital microscopy on the thin film surfaces (island/hole structures) and SAXS in the bulk samples (Figure S7 and S8).

### MC to SP under visible light

**Figure 7.**
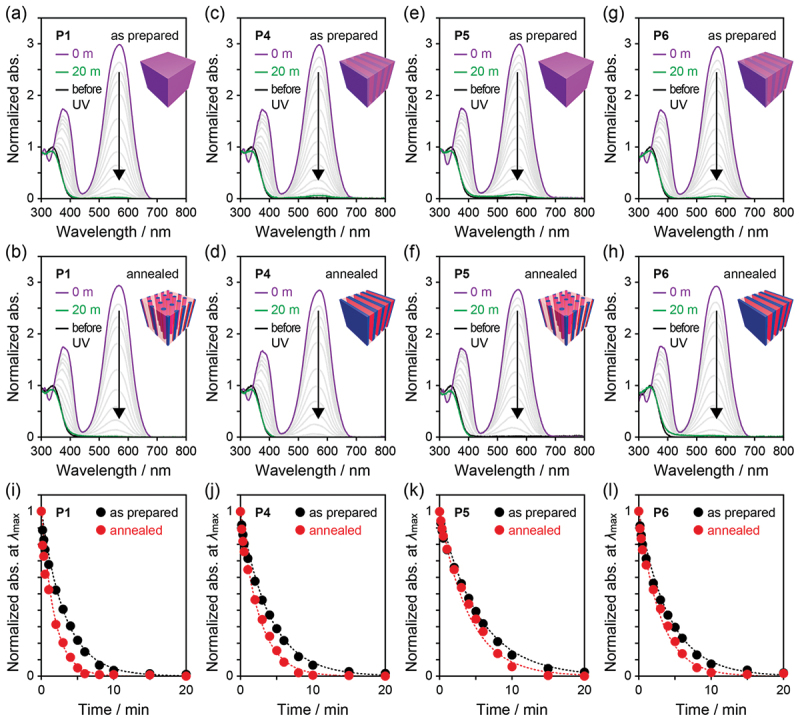
UV–vis absorption spectra of as-prepared and annealed thin films of (a, b) **P1**, (c, d) **P4**, (e, f) **P5**, and (g, h) **P6** under 525 nm irradiation after 365 nm exposure. The spectra are normalized at *λ*_max_ around 340 nm before 365 nm irradiation. (i–l) Time dependence of normalized absorbance at *λ*_max_ above 450 nm of the thin films under 525 nm irradiation.

Next, we exposed the thin films of **P1** and **P4**–**P6** post-irradiated with 365 nm UV light to 525 nm visible light (Figure 7(a–h)). In the UV–vis absorption spectra of the four dBCPs, the MC band gradually decreased with exposure time and eventually disappeared, indicating almost complete isomerization to SP and reversible nature of the incorporated SP molecules. As in the case of SP-to-MC isomerization under UV light, the kinetic curves of the normalized absorbance at approximately 580 nm, plotted as a function of irradiation time, were well described by first-order kinetics (*A*(*t*) = exp(−*kt*)) (Figure 7(i–l) and Table 2) [49–51]. The photoisomerization rate were faster in the ordered (annealed) films than in the disordered (as-prepared) films probably due to the enhanced molecular mobility of the low-*T*_g_ P(SPA-*stat*-BA) block and larger local free volume surrounding the MC molecules through distinct segregation from the high-*T*_g_ PMMA block (Figure 6) but independent of the microphase-separated morphology type (cylindrical or lamellar). No clear correlations were found between the *k*s and the compositions or *M*_n_s of the dBCPs. Since the as-prepared and annealed thin films showed similar fluorescence spectra, (negligible) quantum yields, and lifetimes originating from MC molecules after UV light irradiation (Figure S9), no significant differences were observed in their aggregated states. From these results, we concluded that both directions of SP–MC photoisomerization are partially controllable by microphase separation in BCPs.

### Repeatability and thermal isomerization

**Figure 8.**
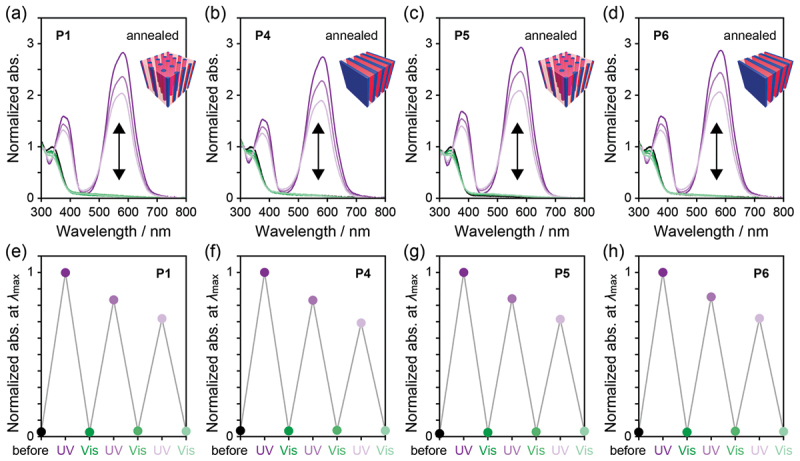
UV–vis absorption spectra of annealed thin films of (a) **P1**, (b) **P4**, (c) **P5**, and (d) **P6** in alternating irradiation with 365 nm and 525 nm. The spectra are Normalized at *λ*_max_ around 340 nm before irradiation. (e–h) Normalized absorbance at *λ*_max_ above 450 nm of the thin films in alternating irradiation with 365 nm and 525 nm.

Repetitive SP–MC photoisomerization was also investigated in the annealed thin films of **P1** and **P4**–**P6** by alternating irradiation with 365 nm UV light for 20 or 30 s and 525 nm visible light for 20 min (Figure 8). In the UV–vis absorption spectra, the two-way photoisomerization was repeatedly observed in three cycles, but the absorption bands of MC at approximately 380 and 580 nm gradually decreased during the cycles due to the H-aggregation and photodegradation.

Additionally, thermal isomerization from MC to SP was examined in the annealed thin films of **P1** and **P4**–**P6** at room temperature in the dark (Figure S10). We confirmed that the incorporated MC molecules gradually and incompletely restored to SP, based on the UV–vis absorption spectra. As in the case of photoisomerization, the kinetic curves obeyed first-order kinetics (*A*(*t*) = exp(−*kt*)) (Figure S10 and Table 2). The rates were significantly slower than those in solution but faster than those in glassy polymer matrices [44], owing to the lower *T*_g_s of the P(SPA-*stat*-BA) block below room temperature. The half relaxation time (*τ*_1/2_)—defined as the time required for the deviation to decrease to half of its initial value during the relaxation process from the nonequilibrium state to equilibrium—of the metastable MC was too fast to be measured (on the order of seconds) in solution, but was several hours in the dBCP thin films and several days in the glassy polymers [44]. No noticeable differences were observed among the dBCPs with the different compositions, *M*_n_s, and morphology types.

## Conclusion

In this study, we synthesized and thoroughly characterized the series of dBCPs (PMMA-*b*-P(SPA-*stat*-BA)) consisting of a high-*T*_g_ PMMA block and a low-*T*_g_ statistical copolymer (P(SPA-*stat*-BA)) block of SPA and BA, with varying compositions, *M*_n_s, and microphase-separated structures. The comparison between the ordered (annealed) and disordered (as-prepared) microphase-separated dBCPs demonstrated that the microphase separation can partially control not the yields but only the rates of both SP-to-MC and MC-to-SP photoisomerization while the ordered morphologies scarcely affected the photoisomerization. These findings provide valuable insights into the molecular and polymer design of photoresponsive smart materials based on photoswitches and will contribute to their further development and applications.

## Supplementary Material

Supplemental Material

## Data Availability

The authors declare that the data supporting the findings of this study are available within the article and its supplementary information file. Additional data may be obtained from the corresponding authors upon request.
