# Supplementary material for: Isomerization of spiropyran photoswitches in microphase-separated block copolymers
Source: Sci Technol Adv Mater. 2025 Nov 20;26(1):2590800. doi: 10.1080/14686996.2025.2590800 (PMC12707078; doi:10.1080/14686996.2025.2590800)
Supplement: Supplemental Material [file TSTA_A_2590800_SM1474.docx]

Supporting Information

**Isomerization of spiropyran photoswitches in microphase-separated block copolymers**

Keiichi Imato*, Koki Momota, Ichiro Imae, Tomoyasu Hirai, and Yousuke Ooyama*

*Applied Chemistry Program, Graduate School of Advanced Science and Engineering, Hiroshima*

*University, 1-4-1 Kagamiyama, Higashihiroshima 739-8527, Japan*

*Department of Applied Chemistry, Faculty of Engineering, Osaka Institute of Technology, 5-16-1 Omiya,*

*Asahi-ku, Osaka 535-8585, Japan*

*E-mail: kimato@hiroshima-u.ac.jp, yooyama@hiroshima-u.ac.jp*

# Table of contents

Materials and measurements S2

Synthesis and characterization of dBCPs S3

Photoisomerization and thermal isomerization S7

Reference S12

# Materials

All solvents and reagents were purchased from FUJIFILM Wako Pure Chemical, Tokyo Chemical Industry, or Sigma-Aldrich. Milli-Q water (resistivity >18 MΩ cm) was prepared using a Merck DirectQ UV 3 water purification system. An SP monomer with a polymerizable acrylate group, **SPA**, was prepared according to our previously reported method [1].

# Measurements

^1^H NMR spectra were recorded at 25 °C using Varian 400 (400 MHz) FT NMR spectrometers in CDCl_3_. Size exclusion chromatography (SEC) measurements were performed at 40 °C using a Shimadzu Prominence-i LC-2030 Plus system equipped with a guard column (LF-G, Shodex), two seriesconnected columns (LF-804, Shodex), a UV detector, and a differential refractive index (RI) detector (RID-20A). THF was used as the eluent, and the SEC system was calibrated with poly(methyl methacrylate) (PMMA) standards. Photoabsorption spectra were recorded at room temperature using a Shimadzu UV-3600i Plus spectrophotometer. Differential scanning calorimetry (DSC) measurements were performed under a nitrogen atmosphere by heating samples three times from −50 °C to 150 °C at a rate of 10 °C min^−1^ using a Hitachi DSC7000X. The glass transition temperatures (*T*_g_s) were determined from the inflection points in the third heating scans. Small angle X-ray scattering (SAXS) measurements were carried out at room temperature on beamline BL40B2 at SPring-8 using incident Xrays with wavelength of *λ* = 0.1 nm. The distance from the sample to the detector (2106 mm) was calibrated with silver behenate. The scattering vector *q* was defined as *q* = 4π sin(*θ*/*λ*), where 2*θ* is the Bragg angle. Surface structures of thin films were observed at room temperature using a Keyence VHX7000 digital microscope. Thickness of thin films was measured at room temperature using a Keyence VK-9700 3D laser microscope. Fluorescence spectra were measured using a HORIBA FluoroMax spectrophotometer. Fluorescence quantum yields were determined using a HORIBA FluoroMax spectrofluorometer with a calibrated integrating sphere system. Fluorescence decay measurements were performed on a HORIBA DeltaFlex modular fluorescence lifetime system using a NanoLED pulsed

diode excitation source (366 nm).

# Synthesis and characterization of dBCPs

Six diblock copolymers (dBCPs) composed of a PMMA block and a statistical copolymer (P(SPA-*stat*BA)) block (PMAA-*b*-P(SPA-*stat*-BA)), **P1**–**P6**, were synthesized by reversible addition–fragmentation chain-transfer (RAFT) polymerization of methyl methacrylate (MMA) and subsequent

copolymerization of **SPA** and *n*-butyl acrylate (BA) (Figure 2).

**Typical procedure for PMMA.** A solution of MMA, 2-cyano-2-propyl dodecyl trithiocarbonate (RAFT agent), and azobisisobutyronitrile (AIBN) in toluene was prepared under a nitrogen atmosphere, purged with nitrogen by bubbling for 15–30 min, and stirred at 65 or 70 °C. After the reaction, the mixture was cooled in an ice bath, diluted with toluene, and precipitated into cold methanol. The precipitate was collected and dried under vacuum to give PMMA as a white solid. ^1^H NMR (400 MHz, CDCl_3_, Figure S1): *δ* (ppm) = 3.60 (br, OCH_3_), 2.08–1.76 (m, CH_2_), 1.49–0.76 (m, CH_3_). Four PMMAs were obtained, and their reaction conditions, yields, number average molecular weights (*M*_n_s), and dispersities (*M*_w_/*M*_n_s) were summarized in Table S1.

**Table S1.** Rection conditions, yields, *M*_n_s, and *M*_w_/*M*_n_s for PMMAs

|  | MMA  / mL (mmol) | RAFT agent / mg (mmol) | AIBN  / mg (μmol) | Toluene / mL | Temp.  / °C | Time  / h | Yield  / g | *M*_n_  / g mol^−1^ | *M*_w_/*M*_n_ |
| --- | --- | --- | --- | --- | --- | --- | --- | --- | --- |
| **PMMA1** | 8.5  (80) | 55  (0.16) | 7.0  (43) | 4 | 70 | 6.5 | 3.5 (44%) | 22000 | 1.19 |
| **PMMA2** | 10  (94) | 77  (0.22) | 3.6  (22) | 55 | 65 | 74 | 4.8 (51%) | 25300 | 1.22 |
| **PMMA3** | 8.5  (80) | 55  (0.16) | 3.0  (18) | 40 | 70 | 30.5 | 4.0 (49%) | 26200 | 1.24 |
| **PMMA4** | 8.5  (80) | 55  (0.16) | 7.0  (43) | 4 | 70 | 22 | 3.8 (47%) | 36100 | 1.21 |


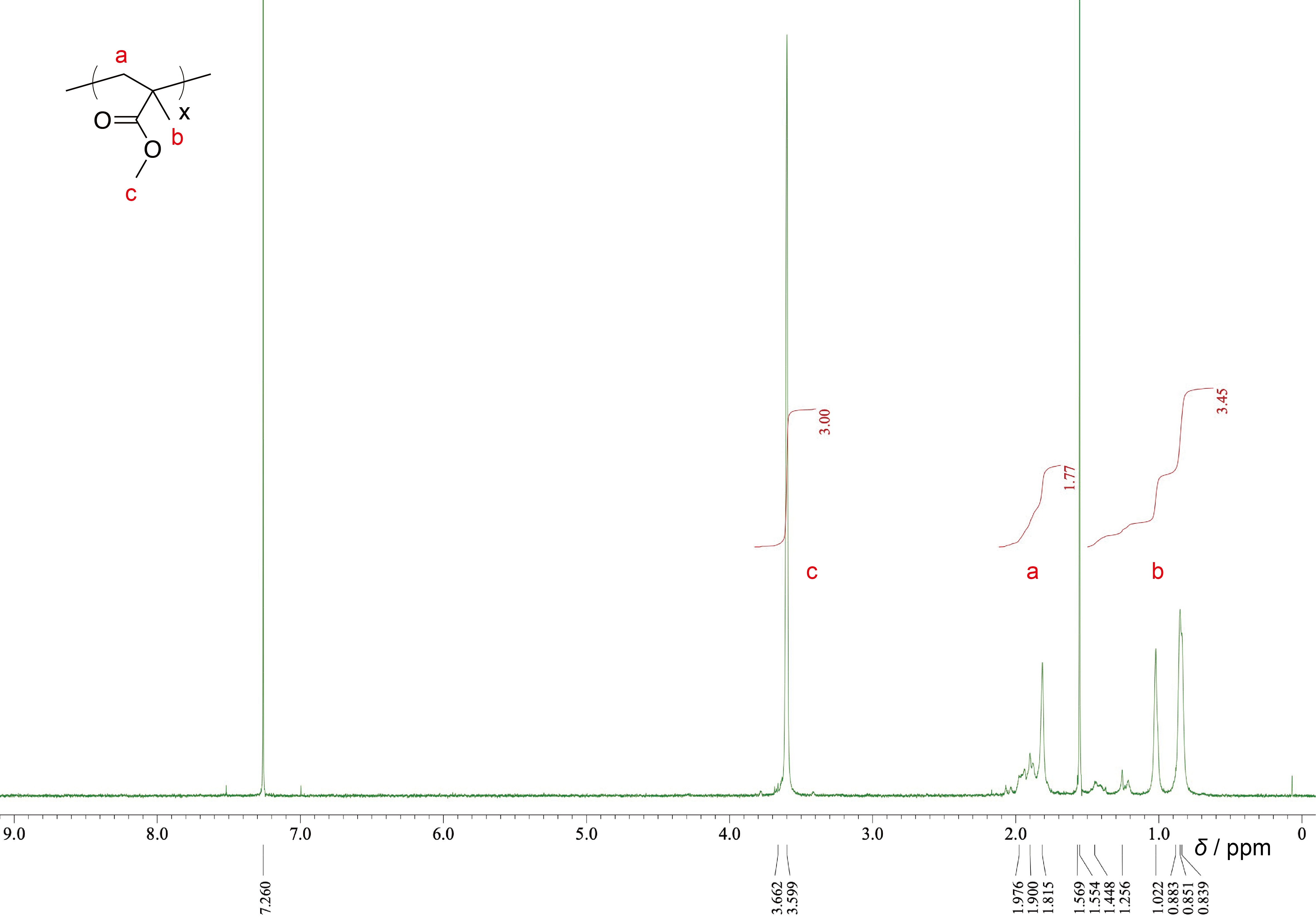


**Figure S1.** ^1^H NMR spectrum (400 MHz, CDCl_3_) of PMMA.


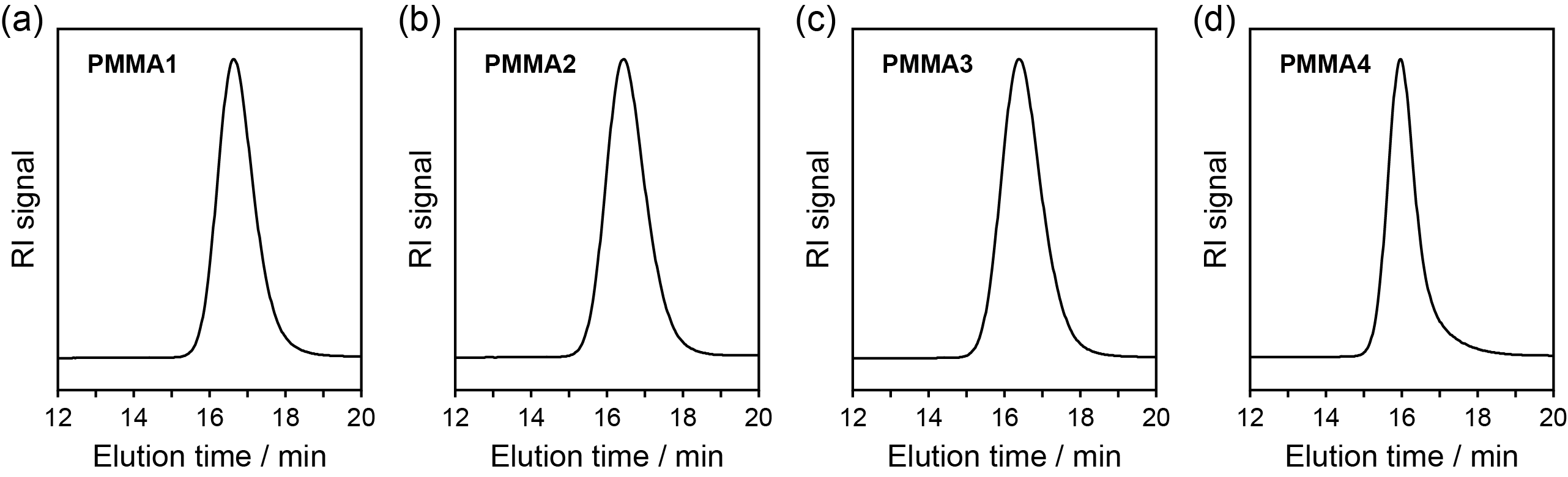


**Figure S2.** SEC curves of (a) **PMMA1**, (b) **PMMA2**, (c) **PMMA3**, and (d) **PMMA4**.

**Typical procedure for PMMA-*b*-P(SPA-*stat*-BA).** A solution of **SPA**, BA, PMMA (RAFT

agent), and 2,2’-azobis(4-methoxy-2,4-dimethylvaleronitrile) (V-70) in 1,4-dioxane was prepared under a nitrogen atmosphere. The solution was deoxygenated via three freeze-pump-thaw cycles and stirred at 30 °C under a nitrogen atmosphere. After the reaction, the mixture was cooled to room temperature, diluted with 1,4-dioxane or dichloromethane, and precipitated into cold methanol. The precipitate was collected and dried under vacuum to give PMMA-*b*-P(SPA-*stat*-BA) as a white solid. ^1^H NMR (400 MHz, CDCl_3_, Figure S3): *δ* (ppm) = 7.99 (br, aromatic), 7.20–6.60 (m, aromatic and =CH–), 5.88 (br, =CH–), 4.20–3.95 (m, OCH_2_), 3.50–3.30 (m, OCH_3_ and NCH_2_), 2.26 (br, CH), 2.08–1.70 (m, CH_2_),

1.70–0.70 (m, CH_2_ and CH_3_). The reaction conditions, yields, *M*_n_s, and *M*_w_/*M*_n_s were summarized in Table S2.

**Table S2.** Rection conditions, yields, *M*_n_s, and *M*_w_/*M*_n_s for PMMA-*b*-P(SPA-*stat*-BA)s

|  | **SPA** BA  / g (mmol) / mL (mmol) | | PMMA  / g (μmol) | V-70 / mg (μmol) | 1,4-Dioxane Temp.  / mL / °C | | Time | Yield  / g | *M*_n_  / g mol−1 *M*w/*M*n | |
| --- | --- | --- | --- | --- | --- | --- | --- | --- | --- | --- |
| **P1** | 0.35  (0.86) | 0.56 (3.9) | 0.19 (8.5) | 0.50 (1.6) | 1.5 | 30 | 72 h | 0.49 (47%) | 73900 | 1.36 |
| **P2** | 0.40  (0.98) | 0.64 (4.4) | 0.21 (8.2) | 0.50 (1.6) | 0.7 | 40 | 44 h | 0.48 (41%) | 65200 | 1.33 |
| **P3** | 0.35  (0.86) | 0.59 (4.1) | 0.15 (6.0) | 0.40 (1.3) | 0.5 | 30 | 54 h | 0.38 (37%) | 69200 | 1.42 |
| **P4** | 0.35  (0.86) | 0.57 (4.0) | 0.16 (6.1) | 0.40 (1.3) | 0.5 | 30 | 54 h | 0.38 (37%) | 56000 | 1.33 |
| **P5** | 0.40  (0.98) | 0.64 (4.4) | 0.13 (4.9) | 0.35 (1.1) | 0.5 | 40 | 4 d | n.d. | 67800 | 1.35 |
| **P6** | 0.40  (0.98) | 0.64 (4.4) | 0.22 (6.1) | 0.50 (1.6) | 0.7 | 40 | 44 h | 0.50 (42%) | 88000 | 1.38 |


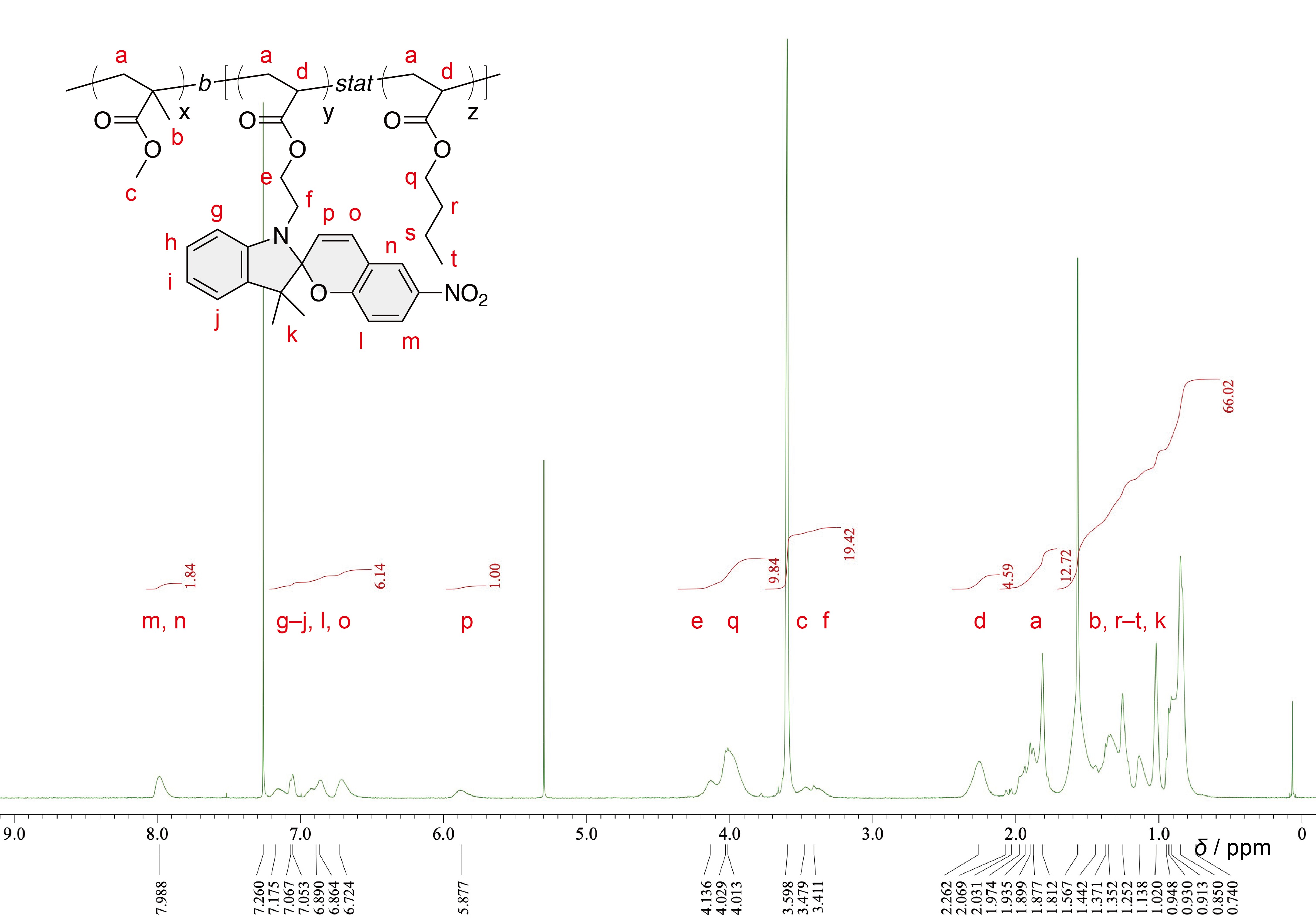


**Figure S3.** ^1^H NMR spectrum (400 MHz, CDCl_3_) of PMMA-*b*-P(SPA-*stat*-BA).


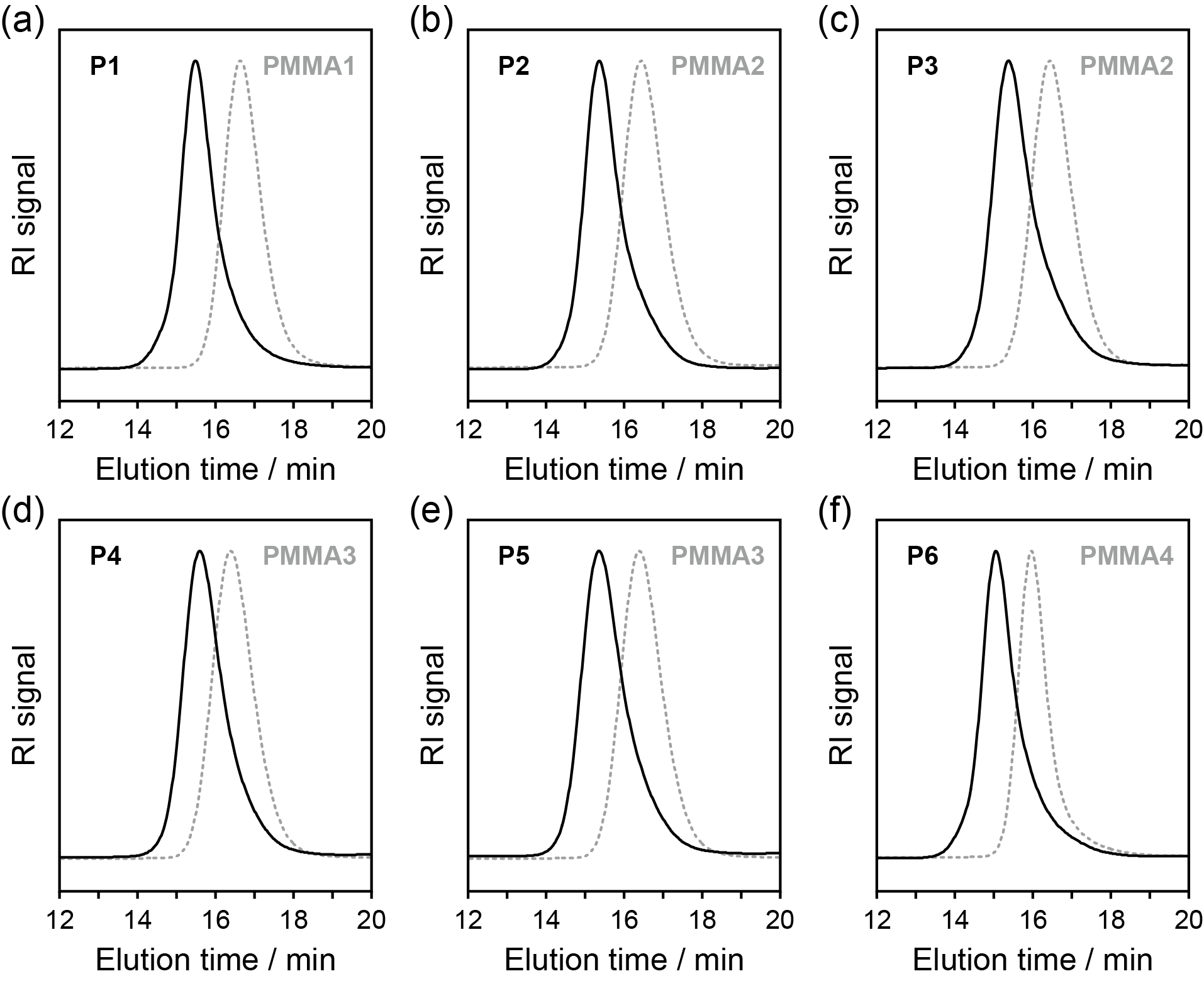


**Figure S4.** SEC curves of (a) **P1**, (b) **P2**, (c) **P3**, (d) **P4**, (e) **P5**, and (f) **P6**.

# Photoisomerization and thermal isomerization

Photoisomerization and thermal isomerization of four dBCPs, **P1** and **P4**–**P6**, were investigated by UV– vis absorption spectroscopy on the thin films before and after annealing at 180 °C for 16 h under vacuum. The thin films were prepared by the following procedure. First, glass substrates were treated with piranha solution to clean the surfaces and expose hydroxyl groups, washed with water, and dried under vacuum at room temperature. Then, the hydrophilic substrates were treated with 1,1,1,3,3,3hexamethyldisilazane (HMDS) at 140 °C for 15 min to modify a hydrophobic monolayer, washed by sonication in acetone for 15 min, and dried under vacuum at room temperature. The thin films were obtained by spin coating of the toluene solutions (20 mg mL^−1^) onto the HMDS-modified substrates under a nitrogen atmosphere at 1000 rpm for 30 s using a Mikasa MS-A100 Opticoat spin coater, followed by drying under vacuum at room temperature. LED light sources with a peak wavelength of

365 nm (21.5 mW cm^−2^, LDR2-100UV2-365-W, CCS) and 525 nm (7.74 mW cm^−2^, LDR2-90GR2, CCS) were employed for the photoisomerization experiments (Figure S5). The exposure distance was 40 mm for the 365 nm light and 20 mm for the 525 nm light.


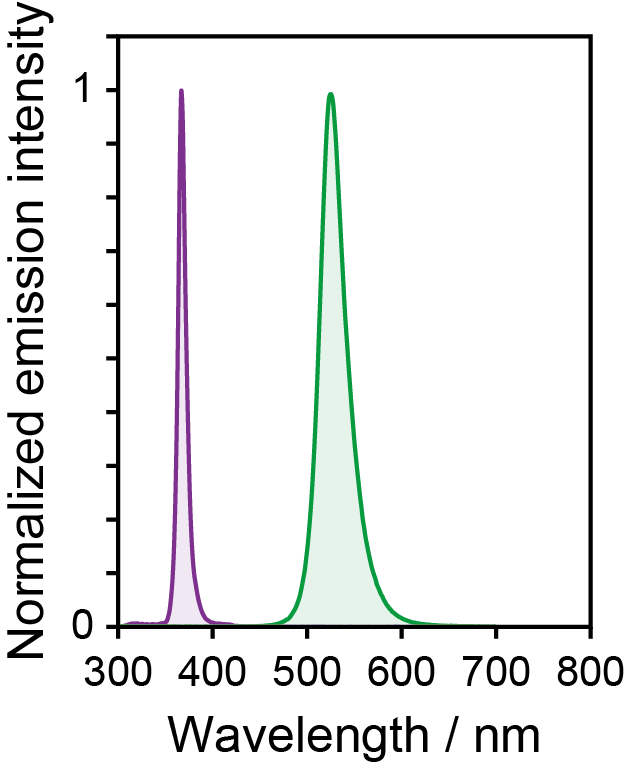


**Figure S5.** Normalized emission spectra of 365 nm and 525 nm LED light.


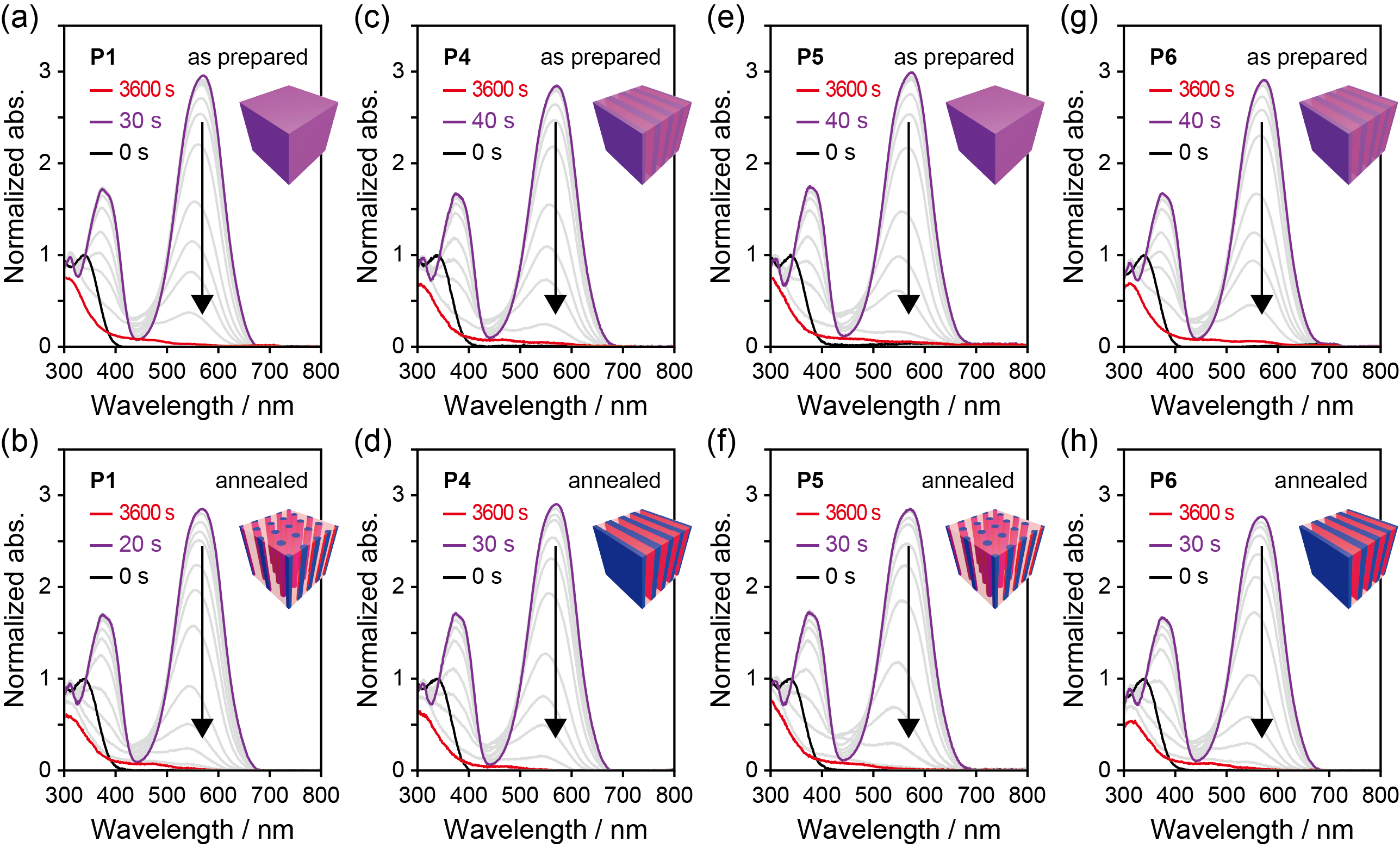


**Figure S6.** UV–vis absorption spectra of as-prepared and annealed thin films of (a, b) **P1**, (c, d) **P4**, (e, f) **P5**, and (g, h) **P6** under excessive 365 nm irradiation. The spectra are normalized at *λ*_max_ around 340 nm before irradiation.


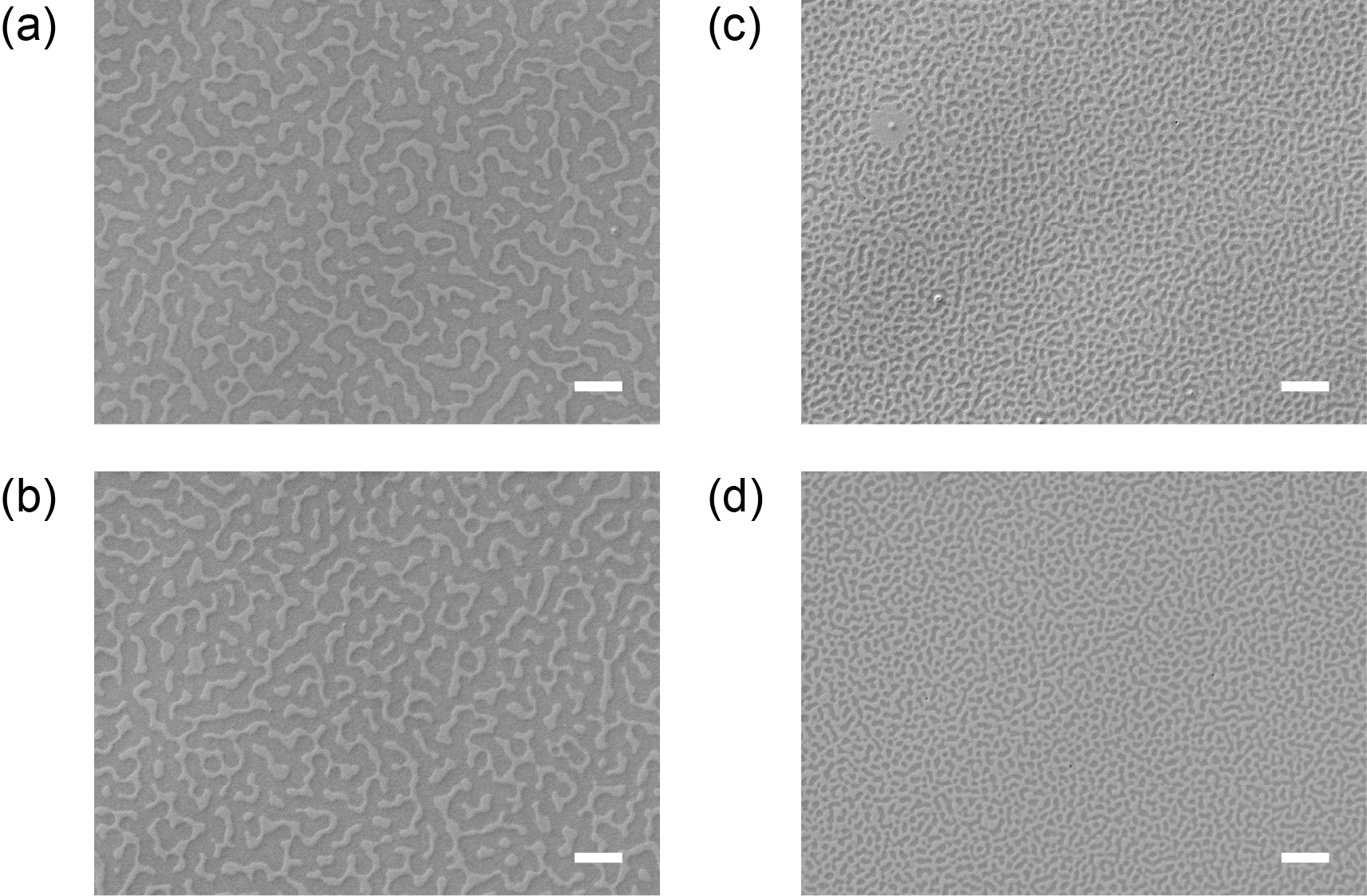


**Figure S7.** Digital microscopic images of annealed thin film surfaces of **P1** (a) before and (b) after 365 nm irradiation and **P6** (c) before and (d) after 365 nm irradiation.


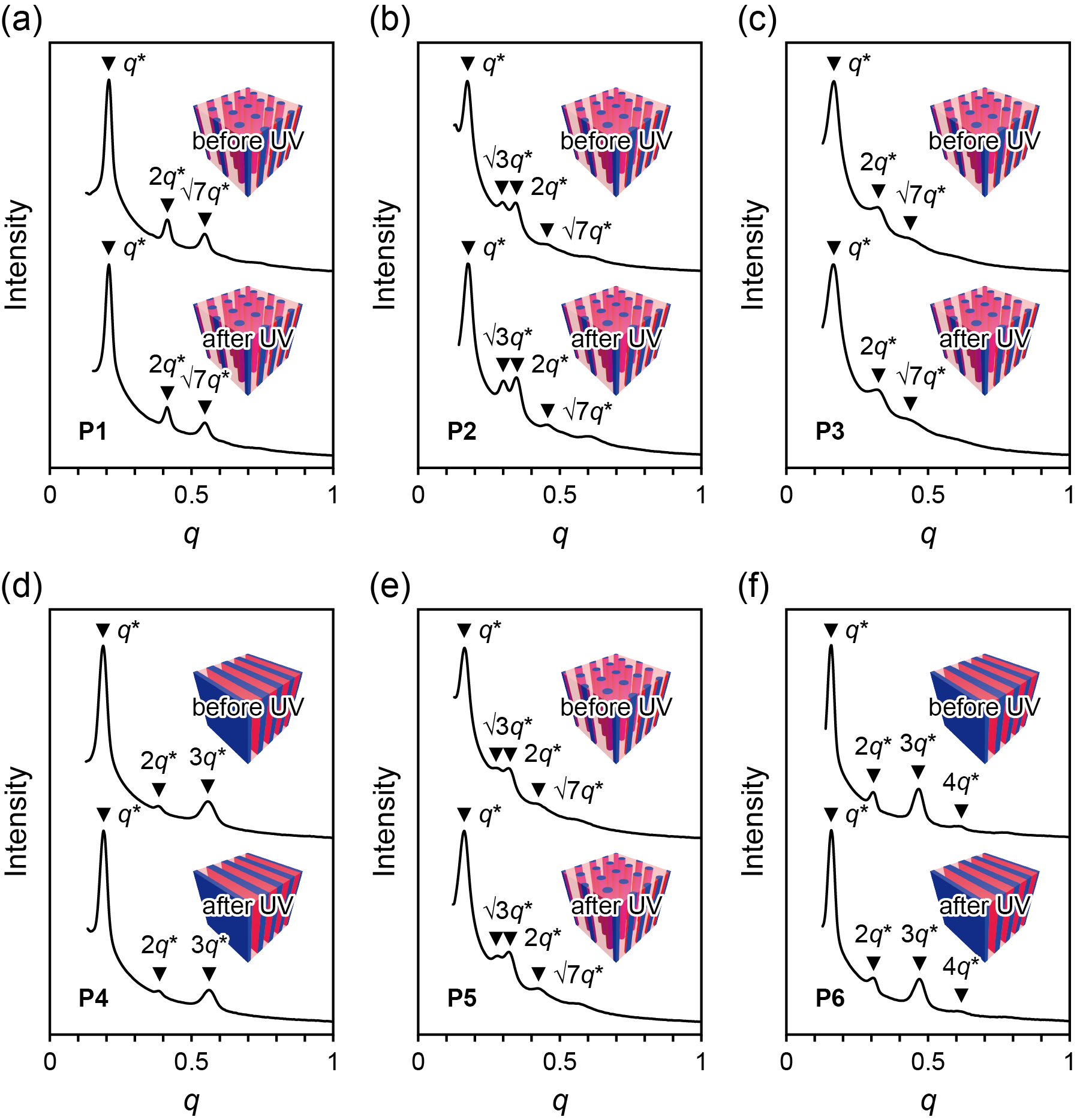


**Figure S8.** SAXS patterns of annealed (a) **P1**, (b) **P2**, (c) **P3**, (d) **P4**, (e) **P5**, and (f) **P6** before and after 365 nm irradiation.


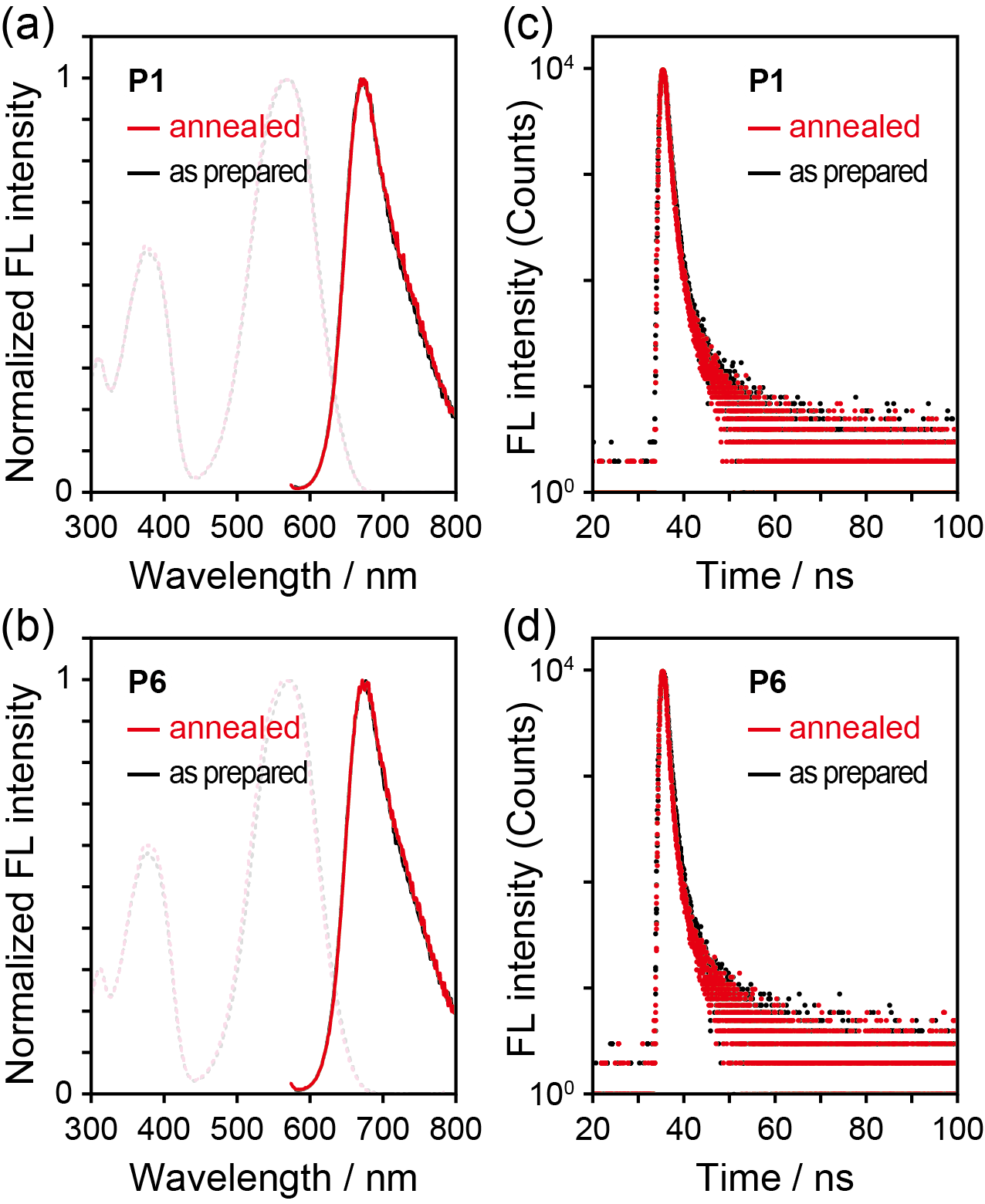


**Figure S9.** Fluorescence (FL) spectra (*λ*_ex_ = 564 nm) of as-prepared and annealed thin films of (a) **P1** and (b) **P6**. FL decay profiles (*λ*_ex_ = 366 nm, *λ*_em_ = ca. 670 nm) of as-prepared and annealed thin films of (c) **P1** and (d) **P6**.


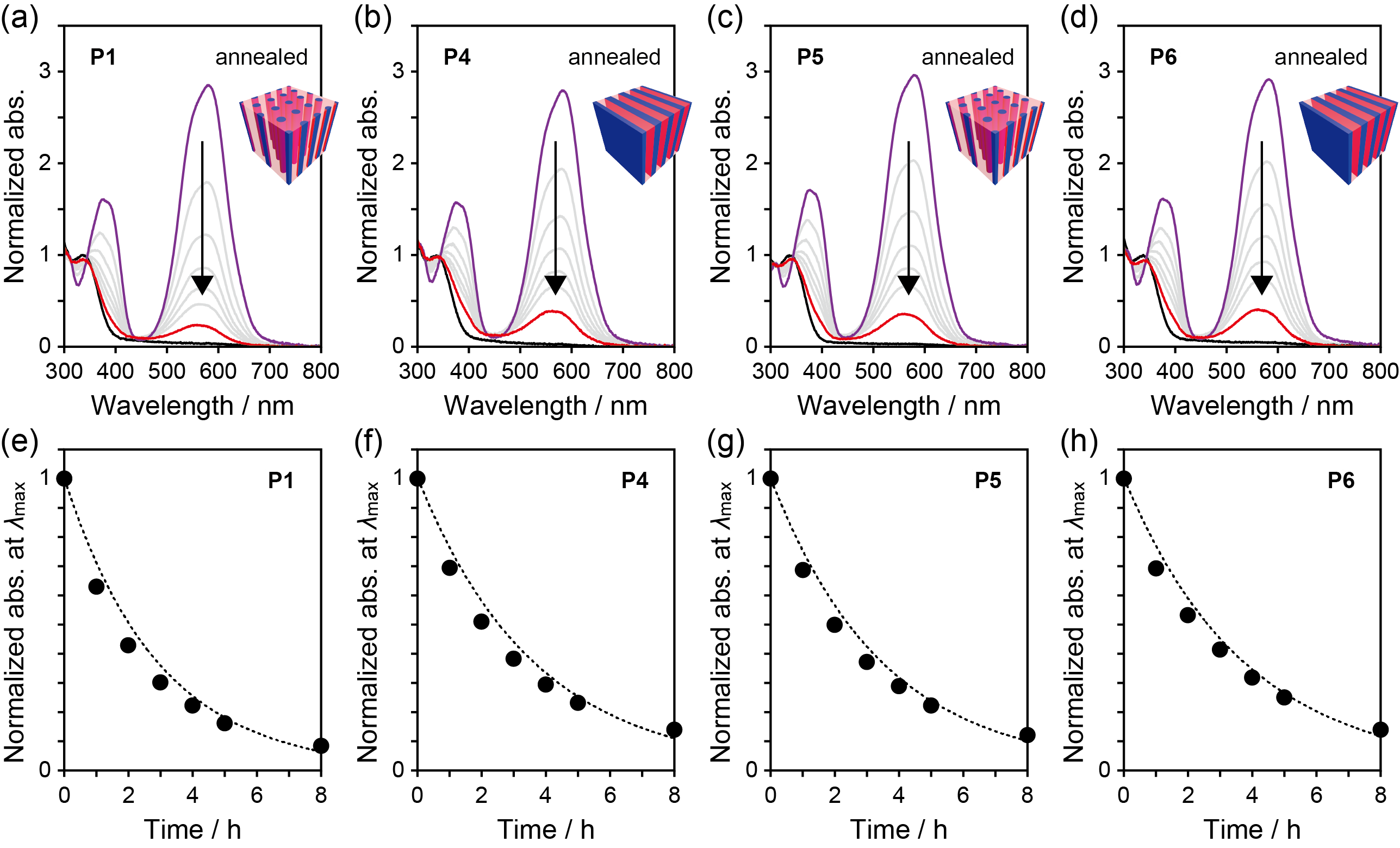


**Figure S10.** UV–vis absorption spectra of annealed thin films of (a) **P1**, (b) **P4**, (c) **P5**, and (d) **P6** during thermal isomerization at room temperature in the dark after 365 nm exposure. The spectra are normalized at *λ*_max_ around 340 nm before 365 nm irradiation. (e–f) Time dependence of normalized absorbance at *λ*_max_ above 450 nm in the thermal isomerization.

# Reference

[1] Imato K, Momota K, Kaneda N, Imae I, Ooyama Y. Photoswitchable adhesives of spiropyran polymers. Chem. Mater. 2022;34(18):8289–8296. doi: 10.1021/acs.chemmater.2c01809
